# On the Surgery of the Hand

**Published:** 1868-05

**Authors:** Stephen Smith

**Affiliations:** Surgeon to Bellevue Hospital.


### SELECTED ARTICLES.

---

### ARTICLE III.

### *On the Surgery of the Hand.*

By Stephen Smith, M.D., Surgeon to Bellevue Hospital.

At our last meeting I called your attention to the progressive developement of the bony skeleton of the fore limb of the mammalia until the act culminated in the human hand. You noticed how the function became more and more complex in the direct ratio to the multiplication of parts. First, it was found as a single digit, and used only in progression ; then a second perfect digit was supplied, and to locomotion was added the function of prehension ; the addition of four fingers renders this function more perfect ; the rudimentary thumb gives the additional power of grasping, at first very imperfect, but increasing in extent and perfection as this digit is lengthened until it attains it highest expression in the long and very movable thumb of the human hand.

Let us now study briefly the movements of the hand and the general arrangement of its muscular forces.

First, there is flexion and extension and lateral motions of the hand as a whole. Flexion and extension of the hand are accomplished by two flexors and three extensors. These muscles are so arranged as to give a radial and ulnar flexor, and two radial and one ulnar extensor. Their relations to the carpus are not intimate though they are called carpal flexors and extensors. Their insertions are, for the most part, into the bases of the metacarpal bones of the index, second, and little fingers, and hence their action must be that of metacarpal rather than carpal flexors and extensors. The lateral movements of the hand are principally affected by the action of the flexors and extensors, on either border acting together.

Second, the four fingers have flexion, extension, abduction and adduction.

Flexion is accomplished by two powerful muscles, the tendons of which are inserted into the bases of the second and third phalanges. Extension is effected by a single muscle, whose tendon is so divided as to be inserted into the bases of both the second and third phalanges. Abduction and adduction is performed by the interossei, which, taking their origin between the metacarpal bones, are inserted into the lateral surfaces of the bases of the first phalanges.

Exceptional fingers are the index and little fingers. The index finger has an additional extensor muscle inserted with the common extensor, while the little finger has an abductor on its ulnar border, a short flexor inserted into the base of the first phalangeal bone on the external surface, and a flexor of its metacarpal bone. This latter muscle differs from the other muscles which we have met in location and function. It has its origin from the ulnar side of the central region of the carpus, and is spread out at its insertion so as to grasp the whole extent of the metacarpal bone of the little finger on its external aspect. This bone is much more movable at its articulation with the carpus than the other metacarpal bones of the fingers. The flexor digiti minimi has, therefore, three actions. First, it is a flexor of the metacarpal bone of the little finger. Second, it rotates the metacarpal bone towards the palm. Third, in these combined acts we notice the third, and doubtless the most important function, the approximation of the metacarpal bone of the little finger to the thumb. This muscle, often called the opponens digiti minimi, adds much to the power of grasping.

The first digit, or thumb, may well be considered as an independent extremity. Its bony skeleton is unique in that it is deficient by one phalangeal bone ; its method of articulation gives it the freedom of motion peculiar to enarthrodial joints ; and, finally, its supply of muscles is special and independent of the other fingers.

The motions of the thumb are flexion, extension, abduction, adduction, opposition and circumduction. Flexion is effected by three muscles of great power, but of unequal size. The long flexor, having an extensive origin in the forearm, is inserted into the base of the last phalangeal bone, its tendon passing between the heads and tendons of the short flexor, somewhat as the tendons of the flexor profundus pass between the divided tendons of the flexor sublimis. The short flexor has an extensive origin from the central region of the carpus ; its fibres converging from two separate tendons which are inserted into the base of the first phalanx, on their respective sides. Each tendon has developed within it, over the articulation, a sesamoid bone.

The flexor ossis metacarpi pollicis differs from the two preceding muscles in having a small origin and a very extensive insertion. It arises at the base of the thumb, from the trapezium, and is inserted into the entire extent of the metacarpal bone of the thumb, along its radial border. The insertion upon the external aspect of the metacarpal bone gives an addition to the function of flexion, the powers of opposing the palmar surface of the thumb to the palm of the hand, and hence this muscle is often called the opponens pollicis.

Extension of the thumb is effected by three separate muscles, which, arising from the posterior part of the forearm, are inserted into the base of the metacarpal and phalangeal bones, respectively. The relation of these muscles is such that the entire thumb may be extended, and the breadth of the hand greatly increased. This change in the breadth of the hand enlarges its grasp. The importance of a separate extensor muscle for each bone of the thumb is seen in the various adaptations of the thumb to the palm, and the surface and extremities of the fingers, and to objects which are grasped and handled.

Abduction is effected by a small muscle on its radial border, inserted into the base of the first phalanx, and adduction by a powerful muscles arising from the second metacarpal bone and inserted into the inner side of the base of the first phalanx. These muscles are inserted in common with the tendons of the short flexor.

Circumduction, or the rotary motion of the thumb, is effected by the combined action of the muscles now given. The common insertion of the tendons of the abductor and the radial portion of the short flexor, and of the adductor and the ulnar portion of the same muscle, gives an almost continuous stratum of muscular fibre around the palmar portion of the thumb, while the divergent course of the extensor muscles from the several points of insertion so far complete the circle as to render it possible to combine muscular action around the entire organ.

From this brief review of the situation and action of the muscles of the hand, the following general statements may be made:

1. The carpal flexors and extensors are inserted almost exclusively into the base of metacarpal bones.

2. The flexors and extensors of the fingers are inserted into the base of the second and third phalanges.

3. Adductors and abductors are inserted into the lateral surfaces of the bases of the first phalanges.

4. The little finger has an additional muscle, namely, a flexor of its metacarpal, inserted into the entire length of that bone.

5. The thumb has three flexors, and three extensors, viz. one for each bone in its skeleton; they are all inserted into the bases of these bones, except the first flexor, which is attached to the whole length of the metacarpal bone.

6. The thumb has an abductor and an adductor, both of which are inserted into the base of the first phalanx.

7. The flexors are, in general, much the more powerful muscles, owing to the greater power which they must exert in the ordinary function of the hand.—*Medical Gazette.*

